# The Role of IL-15 Deficiency in the Pathogenesis of Virus-Induced Asthma Exacerbations

**DOI:** 10.1371/journal.ppat.1002114

**Published:** 2011-07-14

**Authors:** Vasile Laza-Stanca, Simon D. Message, Michael R. Edwards, Hayley L. Parker, Mihnea T. Zdrenghea, Tatiana Kebadze, Onn M. Kon, Patrick Mallia, Luminita A. Stanciu, Sebastian L. Johnston

**Affiliations:** 1 Department of Respiratory Medicine, National Heart and Lung Institute, MRC & Asthma UK Centre in Allergic Mechanisms of Asthma, Centre for Respiratory Infection, Imperial College London, London, United Kingdom; 2 Imperial College Healthcare NHS Trust, London, United Kingdom; University of Southern California School of Medicine, United States of America

## Abstract

Rhinovirus infections are the major cause of asthma exacerbations. We hypothesised that IL-15, a cytokine implicated in innate and acquired antiviral immunity, may be deficient in asthma and important in the pathogenesis of asthma exacerbations. We investigated regulation of IL-15 induction by rhinovirus in human macrophages *in vitro*, IL-15 levels in bronchoalveolar lavage (BAL) fluid and IL-15 induction by rhinovirus in BAL macrophages from asthmatic and control subjects, and related these to outcomes of infection *in vivo*. Rhinovirus induced IL-15 in macrophages was replication-, NF-*κ*B- and α/β interferon-dependent. BAL macrophage IL-15 induction by rhinovirus was impaired in asthmatics and inversely related to lower respiratory symptom severity during experimental rhinovirus infection. IL-15 levels in BAL fluid were also decreased in asthmatics and inversely related with airway hyperresponsiveness and with virus load during *in vivo* rhinovirus infection. Deficient IL-15 production in asthma may be important in the pathogenesis of asthma exacerbations.

## Author Summary

We previously reported deficiency in interferon production in asthma, which correlated with disease severity and viral load during experimental rhinovirus infection. Here we show that macrophages produce IL-15 upon rhinovirus infection and that IFN-β plays an important role in IL-15 production. In asthmatic subjects, there is a deficiency in rhinovirus-induced production of IL-15 by macrophages, which indicates immunodeficiency in asthma is surprisingly broad, also involving IL-15, an important cytokine that bridges innate and acquired immunity. These results show that IFN-β therapy in asthma exacerbations could be effective not only due to direct anti-viral effects of IFN-β, but also by inducing IL-15 production. We also show induction of IFN-β and IL-15 to be NF-*k*B dependent, an important finding which has implications for NF-*k*B inhibitor drug development programmes as these drugs have potential to worsen rather than improve asthma exacerbation severity, by further enhancing deficiencies of IL-15 and IFN-β. This study investigating the role of IL-15 in rhinovirus infection and asthma has also major implications in other diseases, for example pandemic influenza, where asthma is a major risk factor for severe disease and death, and COPD and cystic fibrosis where IFN-β deficiency is also present.

## Introduction

Rhinovirus (RV) infections in healthy individuals manifest as common colds but in asthma are strongly associated with acute exacerbations [1], [2]. Type I (α/β), II (γ) and III (λ) interferon (IFN) responses are important in anti-viral immunity and increased susceptibility to RV infection has been demonstrated in asthma *in vivo* [2], [3]. RV infection of asthmatic bronchial epithelial cells (BEC) *in vitro* resulted in reduced IFN-β and -λ production and increased viral replication [4], [5]. Monocytes/macrophages are also infected and activated by RV [6] and macrophage IFN-λ production is also impaired in asthma and related to asthma exacerbation severity and virus load *in vivo* [5]. Bronchoalveolar lavage (BAL) cell IFN-γ induction by RV is also impaired in asthma and related to exacerbation severity *in vivo* [3].

IL-15 is important in linking innate and adaptive antiviral immune responses, promoting natural killer (NK) and memory CD8 T cell anti-viral immune responses [7], [8] and monocytes/macrophages produce IL-15 in response to virus infections [7]-[10]. There is little reported data on IL-15 in asthma. IL-15 mRNA expression levels in bronchial biopsies in asthma are not increased [11], while protein levels in sputum are undetectable in normal subjects and steroid naïve asthmatics, but detectable in steroid-treated asthmatics [12]. There is a single report that IL-15 gene expression is increased in asthma exacerbations in children [13] but no data on IL-15 in RV infections. Since (i) α/βIFNs are reported to induce IL-15 in dendritic cells and monocytes [14], [15], (ii) RV induction of IFN-β is reported deficient in asthma [4], (iii) IL-15 is important in innate and acquired antiviral immunity and (iv) there is increased susceptibility to RV infection in asthma [1]–[3] we hypothesized that IL-15 production may be deficient in asthma and related to asthma exacerbation pathogenesis.

We have therefore tested the hypotheses that RV infection of macrophages *in vitro* induces IL-15 production and that this is mediated by α/β IFNs and the transcription factor nuclear factor-*κ*B (NF-*κ*B). In addition, we determined whether RV induction of IL-15 *ex vivo* is deficient in macrophages from asthmatic subjects and whether IL-15 levels in BAL fluid are deficient in asthma. We also investigated whether IL-15 deficiency in asthma is related to parameters of severity and virus load during experimental RV16 infection *in vivo*.

## Results

### RV up-regulates IL-15 production in macrophages

**Figure 1:**
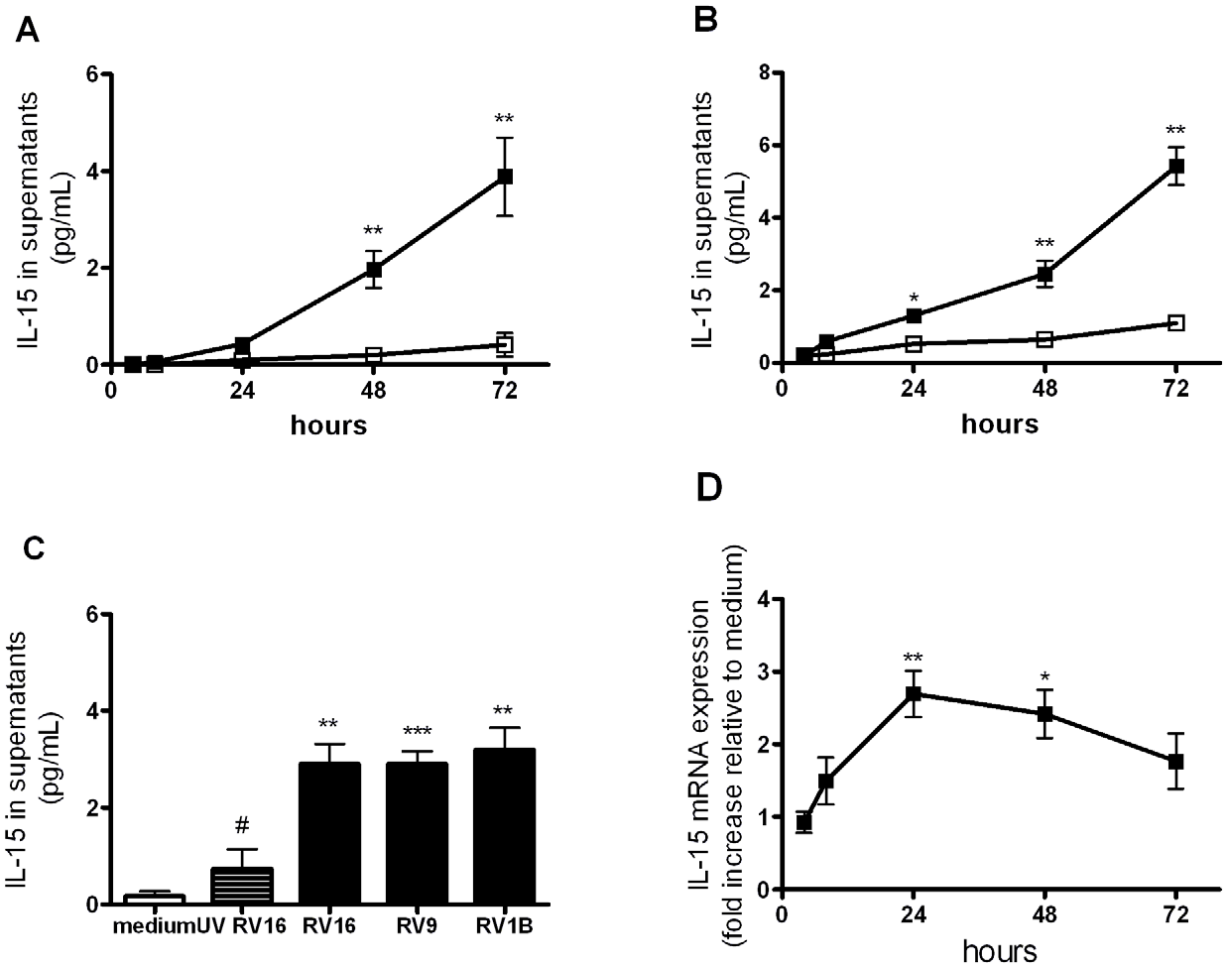
Rhinovirus infection induces IL-15 protein and mRNA production in human monocyte-derived macrophages. (A) THP-1-derived macrophages and (B) peripheral blood monocyte-derived macrophages were infected with RV16 (closed squares) or incubated with medium alone (open squares) at time 0. Supernatants were harvested at 4, 8, 24, 48 and 72 hours and levels of IL-15 released determined by ELISA. (C) THP-1-derived macrophages were exposed to major group rhinovirus (RV16 and RV9), minor group rhinovirus (RV1B), medium alone and UV-inactivated RV16 (UV RV16) at time 0 and supernatants harvested at 72 hours. (D) THP-1-derived macrophages were infected as for (A). Total RNA was extracted from cell lysates at 4, 8, 24, 48 and 72 hours post-infection. IL-15 mRNA was quantified by PCR and results normalised to constitutive 18S ribosomal RNA and expressed as fold induction over medium alone. Mean and SEM from at least four independent experiments (performed in triplicate) are shown. * *P*<0.05, ** *P*<0.01, *** *P*<0.001 for live virus compared to medium, and # *P*<0.05 for live RV16 compared to UV-inactivated.

To investigate whether RV induces IL-15 release from macrophages we used two models of monocyte-derived macrophages in which RV induces replication-dependent activation [6]. We did not measure virus replication in this study however we have previously reported that rhinovirus replication was productive in THP-1 macrophages, leading to release of infectious virus into supernatants, but was limited in monocyte-derived macrophages [6]. IL-15 protein release into supernatants was significantly induced by RV16, at 48 and 72 hours for THP-1-derived macrophages and between 24 and 72 hours for monocyte-derived macrophages (Figure 1A and B). RV9 (major group) and RV1B (minor group) also induced IL-15 release from THP-1-derived macrophages (Figure 1C) indicating that IL-15 induction was not RV serotype- or receptor-dependent. IL-15 release was reduced significantly by UV-inactivation, confirming that induction was largely replication-dependent (Figure 1C).

Because IL-15 protein can be stored intracellularly in macrophages and virus infection could simply trigger the release of preformed protein we next investigated whether RV infection up-regulated IL-15 mRNA expression and found that IL-15 mRNA was significantly induced by RV at 24 hours and 48 hours (Figure 1D).

### NF-*κ*B activation is required for IL-15 induction in RV-infected macrophages

The IL-15 promoter has been shown to have binding sites for nuclear factor (NF)-*κ*B and interferon regulatory factors (IRFs) which are important for its activation by other stimuli [10], [16]–[18]. To assess the role of NF-*κ*B in RV-induced IL-15 production we inhibited activation of the NF-*κ*B pathway with a NF-*κ*B pharmacological inhibitor (AS602868) [6] at the time point of maximal IL-15 production. We used the IKK2 inhibitor AS602868 at a concentration of 5 µM, which we have previously shown to be optimal for rhinovirus induced TNF-α inhibition and without cell toxicity [6].

**Figure 2:**
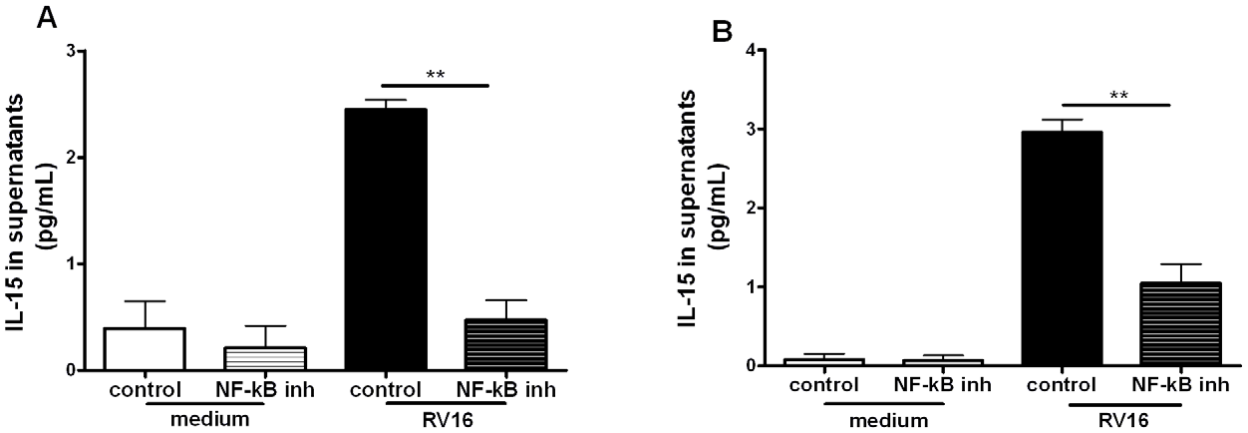
NF-*k*B-activation is required for rhinovirus induction of macrophage IL-15 production. (A) THP-1-derived macrophages and (B) monocyte-derived macrophages were pre-treated for 1 hour with an inhibitor of NF-*κ*B activation (the NF-*κ*B inhibitor AS602868 5 µM) or diluent control, before infection with RV16. The same concentration of drug/diluent was added to the medium after infection. Supernatants were harvested at 72 hours and IL-15 release quantified by ELISA. Means and SEM from at least three independent experiments (performed in triplicate) are shown. ** *P*<0.01 for live virus infected cells, NF-*κ*B inhibitor compared to diluent control.

RV16-induced IL-15 production was significantly decreased in THP-1-derived macrophages (p<0.01) and monocyte-derived macrophages (p<0.01) (Figure 2A and B) in the presence of the NF-*κ*B inhibitor, confirming that RV-induction of IL-15 was NF-*κ*B-dependent in both cell types.

### IFN-α/β induction in RV-infected macrophages is dependent on NF-*κ*B

**Figure 3:**
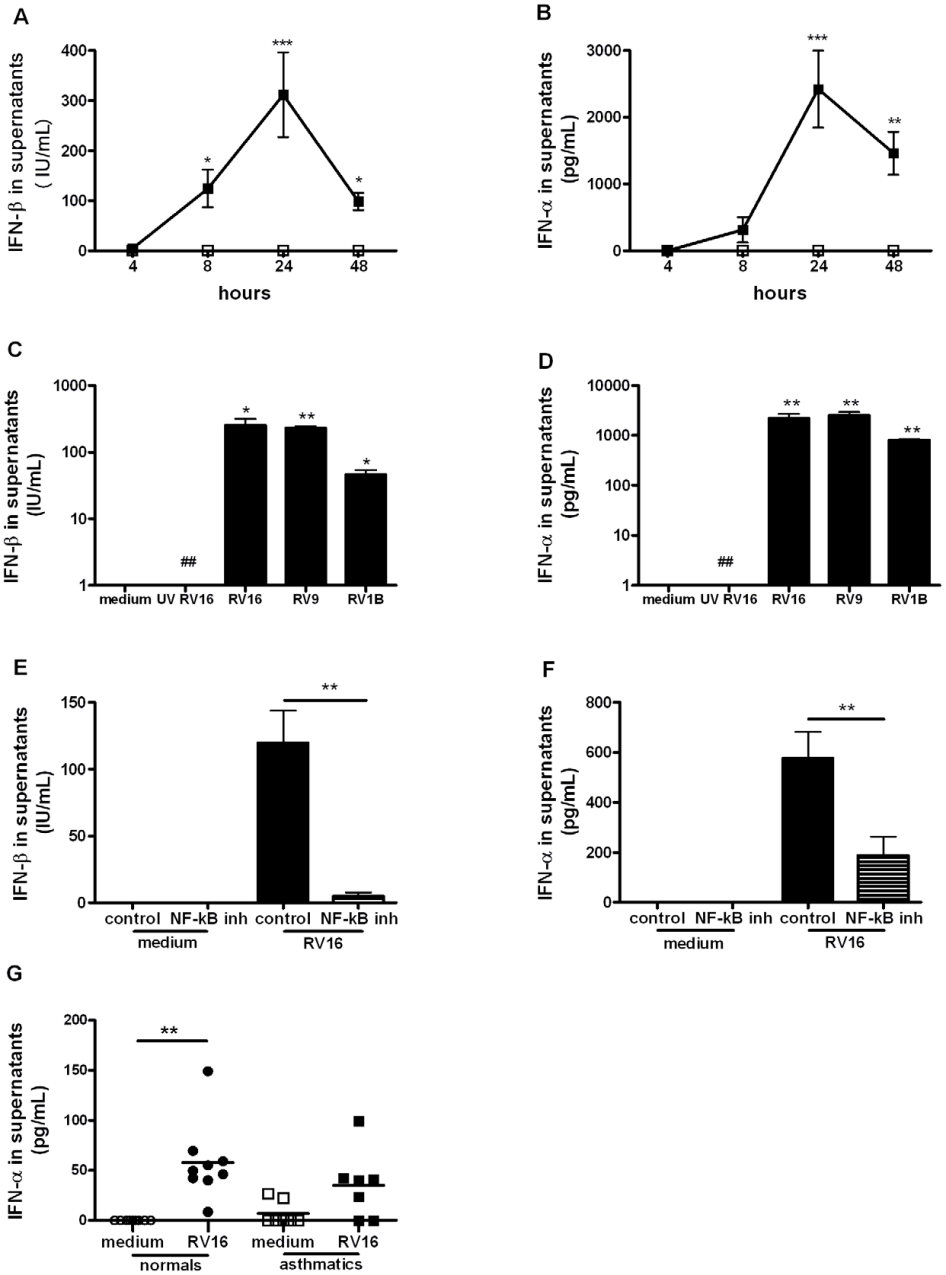
IFN-β and IFN-α are induced by rhinovirus infection of macrophages via NF-*κ*B-dependent mechanisms. (A–B) Monocyte-derived macrophages were infected with RV16 (closed squares) or incubated with medium alone (open squares) at time 0, supernatants were harvested at 4, 8, 24 and 48 hours and levels of IFN-β (A) and IFN-α (B) quantified by ELISA. Means and SEM from at least four independent experiments (performed in triplicate) are shown. (C–D) Monocyte-derived macrophages were exposed to medium alone, UV-inactivated RV16 (UV RV16) or infected with RV16, RV9 or RV1B, cultured for 24 hours, supernatants harvested and IFN-β (C) and IFN-α (D) quantified by ELISA. Means and SEM from at least five independent experiments (performed in triplicate) are shown. *P*<0.05, ** *P*<0.01, *** *P*<0.001 for live virus compared to medium, and ## *P*<0.01 for live RV16 compared to UV-inactivated RV16. (E–F) Monocyte-derived macrophages were pre-treated for 1 hour with an inhibitor of NF-*κ*B activation (the NF-*κ*B inhibitor AS602868 5 µM) or diluent control, before infection with RV16. The same concentration of drug/diluent was added to the medium after infection. Supernatants were harvested at 24 hours and IFN-β (E) and IFN-α (F) quantified by ELISA. Means and SEM from at least five independent experiments (performed in triplicate) are shown. ** *P*<0.01 for live virus infected cells, NF-*κ*B inhibitor compared to diluent control. G. BAL cells from the baseline bronchoscopy of normal (circles, *n* = 9) or asthmatic (squares, *n* = 7) subjects were incubated for 48 hours with medium alone (open symbol, medium) or live rhinovirus (closed symbol, RV16) and IFN-α and IFN-β release into supernatants assessed by ELISA. Bars are median values, ** *P*<0.01, RV16 vs medium.

As α/β IFNs are induced during viral infections and are implicated in IL-15 induction in other systems, we next investigated RV induction of IFN-α/β in macrophages. RV16 infection induced IFN-β production at 8 and 48 hours (p<0.05), peaking at 24 hours (p<0.001, Figure 3A) while IFN-α was induced at 24 hours (p<0.001) and 48 hours (p<0.01, Figure 3B). RV9 and RV1B induced similar levels confirming that IFN-α/β production in macrophages is not serotype- or receptor-dependent and UV-inactivation completely abolished IFN-α/β induction demonstrating that induction is replication-dependent (Figure 3C and D). RV induction of IFN-α/β was also dependent on NF-*κ*B activation as the NF-*κ*B inhibitor markedly decreased RV-induction of IFN-β and IFN-α (Figure 3E and F, both p<0.01).

We previously reported that there is deficient IFN-β production in bronchial epithelial cells in asthmatics upon RV infection [4] and that macrophage production of IFN-λ was similarly deficient [5]. Therefore we wished to investigate if type I IFN production is also deficient in alveolar macrophages from asthmatics. Cells were obtained with bronchoalveolar lavage and the composition of the lavage was ∼90% macrophages in all subjects with no differences in cellular composition between the asthmatics and the non-asthmatics [5]. *In vitro* RV16 infection of BAL cells induced significantly higher IFN-α levels compared with medium in normal subjects (p<0.01) but infection of BAL cells from asthmatics did not result in significantly up-regulated IFN-α levels (Figure 3G). IFN-α levels produced by BAL cells from normals were higher than levels from asthmatics but this difference was not statistically significant (p = 0.09). No IFN-β could be detected in supernatants of BAL cells at the 48 hour time point studied in either normal or asthmatic subjects.

We attempted to confirm deficient production of these IFNs *in vivo*, by measuring IFN-α and IFN-β directly in BAL fluid. Despite using up to 30x concentrated BAL fluid, no IFN-α or IFN-β could be detected in BAL fluid taken at the baseline, day 4 post-RV16 infection or the convalescence bronchoscopies.

### RV induction of IL-15 in macrophages is IFN-α/β dependent

**Figure 4:**
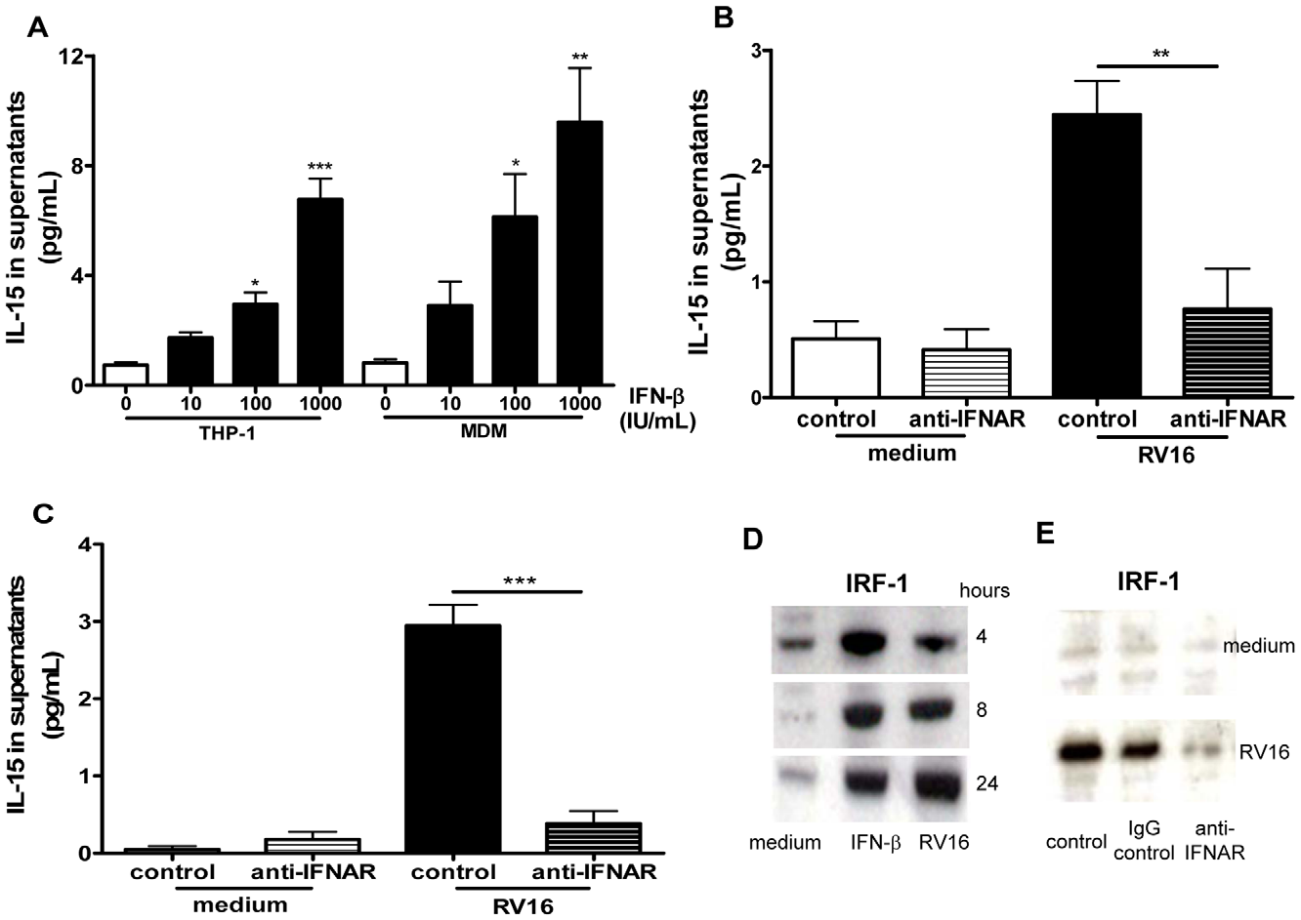
IFN-β induces IL-15 in macrophages and rhinovirus induction is via IFN-αβ receptor signalling. (A) THP-1-derived macrophages (THP-1) or monocyte-derived macrophages were stimulated with diluent control (0), or recombinant IFN-β at concentrations of 10, 100 and 1000 IU/mL, supernatants harvested at 72 hours and IL-15 quantified by ELISA. Means and SEM from at least three independent experiments (performed in triplicate) are shown. * *P*<0.05, ** *P*<0.01 and *** *P*<0.001, compared with diluent control. (B) THP-1-derived macrophages and (C) monocyte-derived macrophages were pre-treated for 1 hour with IFN-αβ receptor blocking antibody (anti-IFNAR) or isotype control (both at 5 µg/mL), the same concentration of antibody was added to the medium after infection with RV16, supernatants were harvested at 24 hours (C) and 72 hours (B) and IL-15 quantified by ELISA. Means and SEM from at least four independent experiments (performed in triplicate) are shown. ** *P*<0.01 and *** *P*<0.001 compared with isotype control. (D) Lysates of THP-1-derived macrophages incubated with medium alone (medium), or stimulated with IFN-β (IFN-β) at 1000 IU/mL or RV16 (RV16) were analysed for the presence of IRF-1 by Western blot at 4, 8 and 24 hours. A representative image of three independent experiments with similar results is shown. (E) Monocyte-derived macrophages treated as in (C) were lysed after 24 hours and analysed for IRF-1 by Western blot. RV16 induction of IRF-1 protein over medium control (medium) was clearly inhibited by prevention of αβ IFN signalling with an IFN-αβ receptor blocking antibody (anti-IFNAR), but not by isotype control (IgG control), or diluent alone (control). A representative image of three independent experiments with similar results is shown.

Having demonstrated that RV induces IL-15 and IFN-α/β production we next investigated whether IFN-α/β signalling is required for RV induction of IL-15 in macrophages. Firstly we investigated whether IFN-β could induce IL-15 secretion in macrophages and found that recombinant human IFN-β induced IL-15 secretion from both THP-1-derived and monocyte-derived macrophages in a dose dependent manner (Figure 4A). Using a blocking antibody against the IFN-α-receptor subunit 2 (IFNAR2) we found that RV16-induced IL-15 production was almost completely inhibited by blocking antibody, but not isotype control in both THP-1-derived and monocyte-derived macrophages (Figure 4B and C, p<0.01 and p<0.001 respectively). Since interferon regulatory factor-1 (IRF-1) is induced by type I IFN [19] and regulates IL-15 gene transcription in other systems [20] we next investigated IRF-1 protein induction by RV. Unstimulated THP-1-derived macrophages expressed low levels of IRF-1, however both IFN-β and RV16 increased IRF-1 intracellular protein levels as early as 4 hours, both remaining elevated to 24 hours (Figure 4D). Blocking the IFNAR, but not isotype control markedly inhibited IRF-1 induction by RV16 (Figure 4E), confirming that the IFN-α/β-IFNAR pathway was also required for IRF-1 activation by RV in macrophages.

### RV induces IL-15 production in alveolar macrophages *ex vivo*; induction is deficient in asthma and related to lower respiratory symptom severity during RV infection

**Figure 5:**
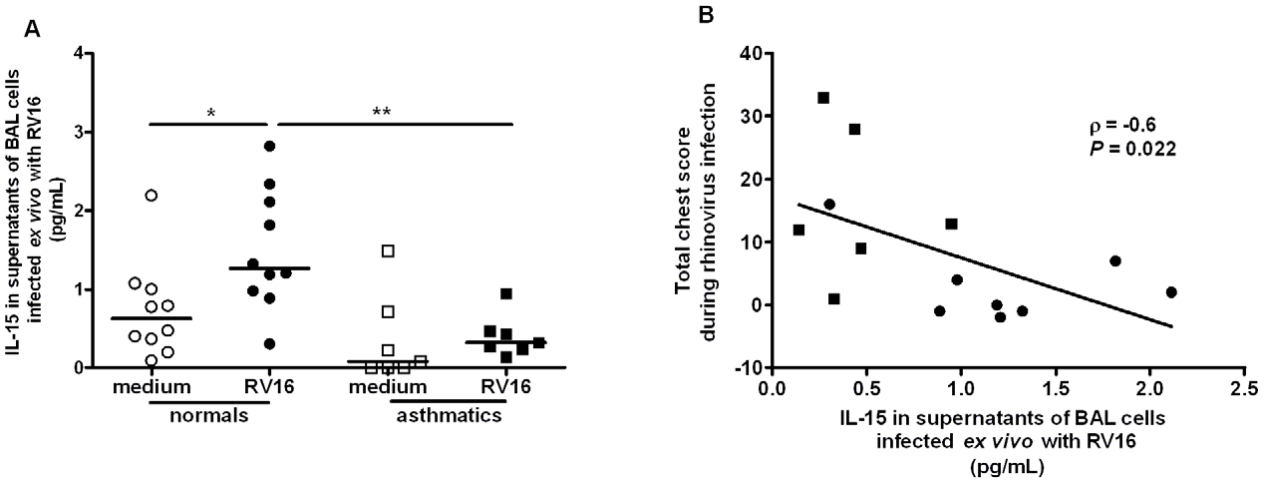
Rhinovirus induction of IL-15 release from alveolar macrophages; induction is inversely related to severity of lower respiratory symptoms following rhinovirus infection *in vivo.* (A) BAL cells were collected at bronchoscopy from normal (circles, *n* = 10) or asthmatic (squares, *n* = 7) subjects. Cells were incubated for 48 hours with medium alone (open symbol, medium) or live rhinovirus (closed symbol, RV16) and IL-15 release into supernatants assessed by ELISA. Bars are median values, * *P*<0.05, RV16 vs medium and ** *P*<0.01 RV16 asthmatic vs. RV16 normal subjects. (B) Levels of IL-15 released *ex vivo* in RV16 infected cultures from normal (closed circles, *n* = 8) and asthmatic (closed squares, *n* = 6) subjects were significantly related to severity of total lower respiratory symptoms during the 2 weeks following experimental infection with RV16 *in vivo*.

Having shown that RV infection induces IL-15 in two macrophage models *in vitro* we next investigated if induction is observed in alveolar macrophage*s* infected *ex vivo* and whether induction is deficient in asthmatics. Supernatants from BAL cells (>90% alveolar macrophages) from normal and asthmatic subjects exposed *ex vivo* to RV16 for 48 hours were assessed for levels of IL-15 by ELISA. IL-15 levels were significantly increased by RV16 in BAL cells from normal subjects (p<0.05), but not in cells obtained from asthmatics, and levels in supernatants from RV16-infected cells were significantly higher in normal compared to asthmatic subjects (Figure 5A, p<0.01). IL-15 production by RV infected macrophages was inversely related to lower respiratory symptom severity on subsequent RV16 experimental infection in the same subjects *in vivo* (r = −0.6, p = 0.022, Figure 5B). Although IL-15 levels were very low, they were above the lower limit of detection (0.25 pg/mL) and differences observed were both statistically significant and related to a number of different clinical parameters.

### BAL IL-15 levels are deficient in asthmatics and related to airway hyperresponsiveness and virus load during subsequent RV infection *in vivo*

To investigate whether IL-15 levels are deficient in asthma *in vivo*, IL-15 was measured in BAL fluid collected from asthmatic and normal subjects when clinically stable prior to experimental RV16 infection. The study recruited 15 non-atopic healthy and 10 atopic mild asthmatic subjects, all rhinovirus-16 serum neutralizing antibody negative and all non-smokers. The healthy control group had a median age of 24, sex ratio 8 male/7 female and a median baseline FEV_1_% predicted of 99% and the asthmatic subjects were inhaled steroid naïve, median age of 22, sex ratio 2 male/8 female and a median baseline FEV_1_% predicted of 104% as previously reported [3].

**Figure 6:**
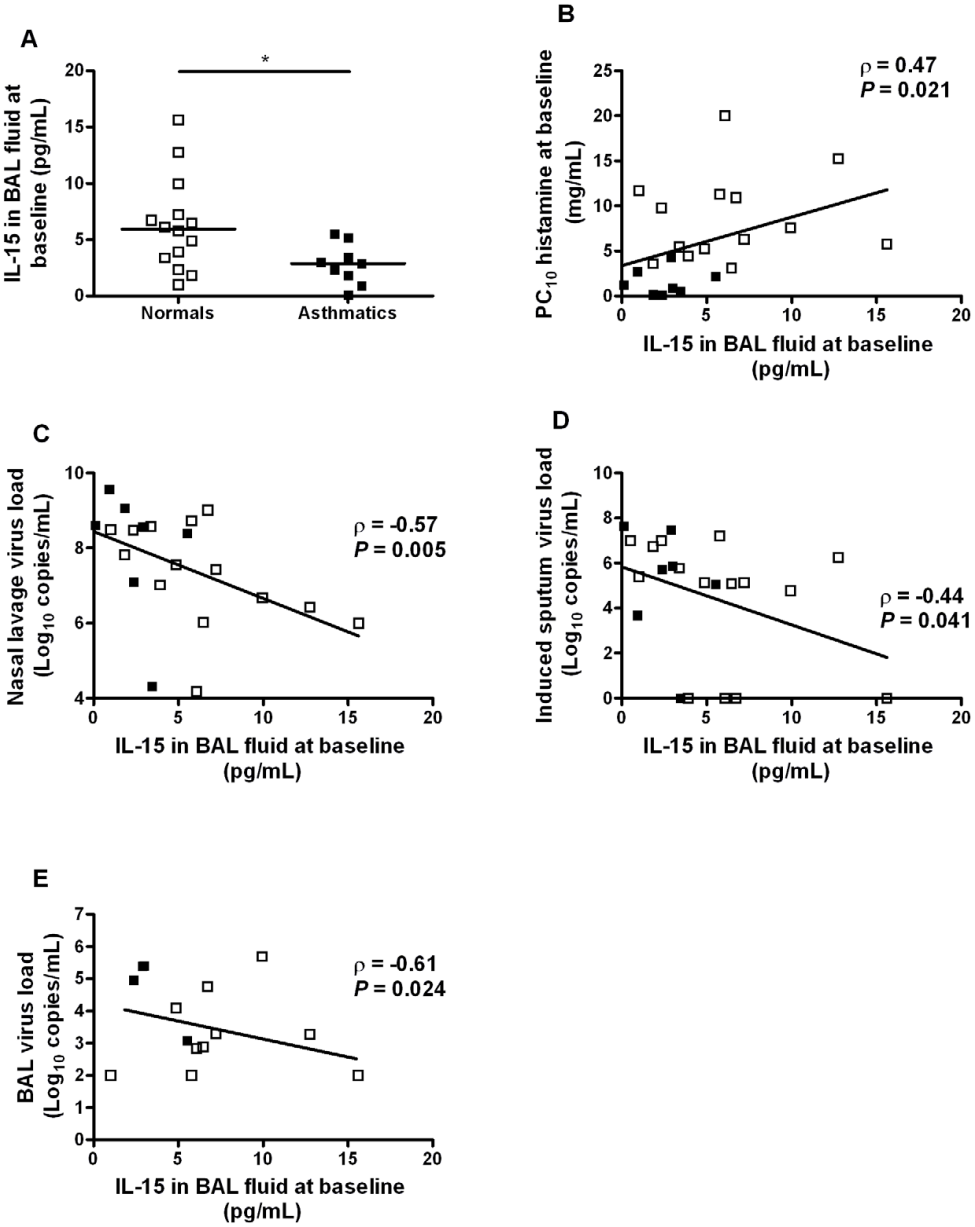
IL-15 levels in BAL fluid in asthma are inversely related to airway hyperresponsiveness and virus load on subsequent *in vivo* rhinovirus infection. Airway hyperresponsiveness was assessed and BAL fluid collected at a baseline bronchoscopy from normal volunteers and asthmatic subjects. Two weeks later, the same subjects were experimentally infected with RV16 and virus load measured in nasal lavage, sputum and BAL. (A) IL-15 levels were measured by ELISA in BAL fluid collected at bronchoscopy from normal (*n* = 14) and asthmatic subjects (*n* = 9). Bars are median values, * *P*<0.05 for comparison between groups. (B) Levels of IL-15 in BAL fluid in asthmatic (*n* = 8) and normal subjects (*n* = 15) were inversely related to bronchial hyperresponsiveness. (C–E) Levels of IL-15 in BAL fluid in asthmatic and normal subjects were inversely related to virus load during a subsequent experimental RV16 infection *in vivo*: (C) peak virus load in nasal lavage, (D) virus load in induced sputum on day 3 post infection, (E) virus load in BAL on day 4 post infection.

IL-15 levels were significantly lower in asthmatic compared to normal subjects (Figure 6A, p<0.05) and were significantly correlated with airway hyperresponsiveness as measured by baseline PC_10_ histamine (r = 0.47, p = 0.021, Figure 6B). IL-15 levels *in vivo* were also significantly related to virus loads in nasal lavage (r = −0.57, p = 0.005), induced sputum (r = −0.44, p = 0.041) and BAL (r = −0.61, r = 0.024) upon subsequent *in vivo* RV16 experimental infection in the same subjects (Figure 6C–E).

## Discussion

We report herein the first investigation of the role of IL-15 in the pathogenesis of RV-induced asthma exacerbations. IL-15 production was induced by RV in macrophage cell lines *in vitro*, as well as in primary alveolar macrophages. IL-15 induction by RV was deficient in cells from asthmatic compared to normal subjects and levels in BAL fluid deficient in asthmatics. IL-15 induction *ex vivo* and levels *in vivo* were both related to airway hyperresponsiveness, lower respiratory symptom severity and virus load following experimental RV16 infection *in vivo*. Finally, IL-15 induction was dependent on IFN-α/β and both IL-15 and IFN-α/β induction were dependent on NF-*κ*B.

These findings have important implications as they suggest IL-15 as a novel candidate for development for treatment or prevention of asthma exacerbations. As IL-15 is deficient in asthma this suggests either or both of prophylactic or therapeutic treatment approaches may have value. Further, IL-15 inducers such as IFN-β may have additional benefit beyond directly enhancing anti-viral immunity in bronchial epithelium, since, via induction of IL-15 production, they may enhance other aspects of both innate and acquired anti-viral immunity. Finally, NF-*κ*B inhibitors are in development as possible therapies for asthma exacerbations, our data that IFN-α, IFN-β and IL-15 induction by RV are all NF-*κ*B dependent suggest that such approaches may further impair already deficient responses in asthma.

Rhinoviruses are the respiratory viruses most commonly associated with asthma exacerbations. Asthma is the most common chronic respiratory disease, and it is increasing in many countries. The major cause of asthma related morbidity and mortality are acute exacerbations and current asthma treatments are only partially effective at preventing asthma exacerbations. Therefore, new approaches to treatment and prevention are urgently required and these are likely to stem from a better understanding of the pathogenesis of asthma exacerbations.

Asthma exacerbation pathogenesis is poorly understood, however, it is clear that people with asthma have increased susceptibility to respiratory virus infections [2], [3]. We have previously reported deficient IFN-β and IFN-λ production in response to RV infection of bronchial epithelial cells in asthmatics [4], [5], further these deficiencies were related to increased RV replication in the same cells, and replacement with exogenous IFN-β restored normal resistance to RV infection. Deficiency of IFN-λ induction by RV was observed in macrophages from asthmatic subjects, and this was related to virus load, asthma exacerbation severity and severity of airway inflammation *in vivo* [5]. These findings led to inhaled IFN-β being developed as a possible novel therapy to treat/prevent asthma exacerbations – currently in a Phase II clinical trial.

We have also recently reported deficient IFN-γ production in asthma and related impaired IFN-γ production to greater virus loads and reductions in lung function in a human model of asthma exacerbation *in vivo* [3]. IFN-γ is produced by both innate and acquired arms of the antiviral immune system. As IL-15 is also important in linking innate and adaptive antiviral immune responses, and in other systems, has been shown to be induced by type I IFNs we wished to investigate the hypothesis that IL-15 might also be deficient in asthma, and that deficiency might be related to the pathogenesis of asthma exacerbations.

Alveolar macrophages are important immune cells in lung antiviral immune responses and we previously reported that monocytes/macrophages support RV replication, become activated and secrete immunomodulatory cytokines [3], [6], [21], [22]. However, there are no published data on IL-15 induction in macrophages by RV, nor on its possible importance in the pathogenesis of asthma exacerbations. Consequently, we investigated macrophage IL-15 and type I IFN responses during RV infection *in vitro* and *in vivo*, in both asthmatic and normal subjects.

The most important findings of this study are the deficient induction of IL-15 by RV in alveolar macrophages from asthmatic subjects *in vitro*, deficient IL-15 levels in asthma in BAL fluid *in vivo*, and the relationships of these to airway hyperresponsiveness, severity of symptoms and virus load on subsequent RV infection *in vivo*. The correlations between IL-15 levels in BAL fluid at baseline and PC_10_ histamine at baseline and virus load during an experimental rhinovirus infection were only statistically significant when all subjects were included. There were no significant correlations within the group of asthmatic subjects alone, presumably due to the low numbers of asthmatic patients included, and larger studies are required to confirm the link between asthma exacerbation pathogenesis, virus replication and IL-15. However these findings taken together suggest IL-15 as a promising candidate for development as a novel therapy to enhance deficient antiviral immunity in asthma, to correct the increased susceptibility to virus infection present in this condition [2], [3] and in particular as a potential treatment to prevent/treat asthma exacerbations.

IL-15, via the IL-2/IL-15 receptor β-chain and common γ chain receptor, promotes NK cell activation, as well as enhancing memory CD8 T cell antiviral immunity [7], [8], [23]. In addition it enhances type I (α/β) IFN production by dendritic cells and macrophages [24], [25] as well as IFN-γ production by NK and CD8 T cells [7], [8], [26]. Since induction of both IFN types is deficient in asthma and related to asthma exacerbation severity, IL-15 has potential to correct deficiencies present in antiviral immunity in asthma.

Our findings also have important implications for development of type I IFNs as potential therapies for asthma exacerbations, as we show that RV infection of macrophages induces α/β IFNs, that IL-15 is induced by IFN-β stimulation and that RV induction of IL-15 in macrophages is dependent on IFN-α/β receptor signalling. These data suggest that administration of α/β IFNs in asthma is likely to enhance IL-15 baseline levels, as well as enhancing induction upon RV infection, both of which we have shown to be deficient in this report. Type I IFN administration might therefore have benefits well beyond correcting the specific deficit in anti-viral immunity in bronchial epithelial cells, indirectly enhancing deficient IFN-γ production, as well as other NK and CD8 responses via correction of deficient IL-15 production.

These findings also have important implications for development programmes for inhibitors of NF-*κ*B [27]. This transcription factor is implicated in many pro-inflammatory responses linked with the pathogenesis of asthma exacerbations and many pharmaceutical companies have active NF-*κ*B inhibitor development programmes. However we find that RV induction of IFN-α, IFN-β and IL-15 are all profoundly suppressed by inhibition of NF-*κ*B. These data suggest that administration of such inhibitors in asthma, while inhibiting pro-inflammatory mediator production, is likely to further impair deficient type I IFN and IL-15 production, and therefore might have potential to increase rather than decrease severity of virus induced asthma exacerbations. Careful investigation of these outcomes, as well as their relationships with clinical outcomes in human models of RV-induced asthma exacerbations [3] may shed further light on these possibilities.

Because signalling by IL-15 occurs via the IL-2/IL-15 receptor β-chain (CD122) and common γ chain receptor (CD132) expressed primarily by NK and memory CD8 T cells [7], [8], [23], [26], it will be of great interest to determine the levels of these receptors on airway NK and CD8 T cells in virus-induced asthma.

Finally, our findings of deficient IL-15 production in asthma, combined with previous reports of deficient IFN-β [4], IFN-λ [5], IFN-γ and IL-12 [3], [22], suggest complex impairment of anti-viral immune responses in asthma. The mechanisms behind these complex deficiencies clearly require urgent investigation. These findings also have major implications for management of pandemic influenza, where asthma is a major risk factor for severe disease and death [28], as replacing deficient anti-viral immune proteins such as IFN-β and now IL-15, may have therapeutic potential to ameliorate severity of disease and perhaps prevent death.

## Materials and Methods

### Cells, viruses and cell infection/stimulation

HeLa and THP-1 cell lines (ECCC) were cultured in E-MEM and RPMI-1640 (Invitrogen, Paisley, UK) respectively with 10% foetal calf serum (FCS). RV serotype 16, 9 (major group) and 1B (minor group) stocks were prepared and their identities confirmed by neutralisation using serotype-specific antibodies (ATCC), UV-inactivation was performed as previously described [29]. Peripheral blood monocyte-derived macrophages and THP-1-derived macrophages were generated and infected with RV at a multiplicity of infection (MOI) of 1 as previously described [6]. Recombinant human IFN-β (10–1000 IU/mL, R&D, Abingdon, UK) was added to wells. Supernatants, RNA or protein lysates were harvested and stored at −80°C.

### IL-15 mRNA quantification

Total RNA was extracted with RNeasy Kit (Qiagen) and 2 µg was used for cDNA synthesis (Omniscript RT Kit, Qiagen). Quantitative RT-PCR was performed using specific primers and probe for IL-15 (forward GGGAAAGTGATGTTCACCCC, reverse CATCTCCGGACTCAAGTGAAATAA, probe ATCTGGATGCAAAGAATGTGAGGAACTGGA)**.** IL-15 gene expression was normalized to 18S rRNA and presented as fold induction relative to medium [30].

### Ethics statement

Ethics approval (No 99/BA/345) was obtained from St Mary's Local Research Ethics Committee, London, UK. All study participants gave written informed consent.

### Human experimental model of RV-induced asthma exacerbation

The exacerbation model, clinical details including allergy testing and lung function, sampling and analysis are described in detail elsewhere [3]. The study recruited 15 non-atopic healthy control and 10 atopic, inhaled steroid naïve asthmatic subjects; all were non-smokers. Assessment of airway hyperresponsiveness by determination of the provocative concentration of histamine inducing a 10% fall in FEV_1_ (PC_10_ histamine) and BAL were performed at baseline ∼2 weeks prior to experimental RV infection as described [3]. Baseline BAL fluid was collected in a single plastic chamber and transferred immediately to polypropylene tubes on ice, transported to the laboratory, filtered (100 µm), centrifuged at 1500 rpm for 10 min at 4°C, the fluid decanted, aliquoted and stored at −80°C till analysed. Because we could not detect IL-15 in un-concentrated BAL fluid, we concentrated the BAL fluid 30 times as described below. Baseline BAL cells were cultured and exposed to RV16 MOI 5/medium as described [3]. After 48 hours, supernatants were harvested and stored at −80°C.

Virus load on days 1–8 and 11 post-infection for nasal lavage, on day 3 post-infection for sputum and on day 4 post-infection for BAL were determined using quantitative PCR as described [3]. Lower respiratory symptom severity during the two weeks post infection was derived using a chest symptom score as described [3].

### BAL fluid concentration for IL-15 ELISA

IL-15 levels were measured after concentrating the baseline BAL fluid up to 30 times using a centrifugal filter with a nominal molecular weight value of 3000 kD (Millipore, Centriplus YM-3). Values were corrected for variability in dilution during lavage, and during subsequent concentration using total protein concentration of the concentrated BAL (Bradford method, Sigma) [31].

### Western blot for IRF-1 expression

THP-1-derived macrophages were lysed into SDS sample buffer (Invitrogen). Western blot was performed as previously described [6]. After blocking, membranes were incubated with rabbit anti-human IRF-1 (1/500) followed by HRP swine anti-rabbit antibody (1/4000) (AbD Serotec). Bands were visualized by chemiluminescence with the ECL Western blotting detection reagent (GE Healthcare).

### IL-15, IFN-α and IFN-β ELISA

Levels of IFN-α and IFN-β were measured using ELISA kits (BioSource International) (sensitivity 15 pg/mL and 5 IU/mL respectively). For the IL-15 ELISA we used commercially available paired antibodies (R&D Systems) at concentrations recommended by the manufacturer and for the standard curve recombinant human IL-15 (Biosource). The protocol recommended by the manufacturer was modified by incubating the plates for 10 minutes with streptavidin-HRP (Biosource) at a concentration of 0.5 µg/mL. A TMB containing substrate solution was used to develop the colour. Once optimised, we could reproducibly detect levels of IL-15 over 0.25 pg/mL.

### NF-*κ*B and IFN - IFN-receptor pathway inhibition

The effect of NF-*κ*B activation on IL-15 production was evaluated using a NF-*κ*B inhibitor (AS602868) as previously described [6]. The role of type I interferons in IL-15 production was assessed using a blocking antibody to IFNAR2, a matched isotype antibody was used as control (Merck Chemicals). Cells were pre-treated for 1 hour before infection with IFNAR2 blocking antibody/isotype control at a concentration of 5 µg/mL. The same concentration of antibody was added to the medium after infection.

### Statistical analysis

The results were analyzed using GraphPad Prism version 4.00 for Windows (GraphPad Software, California, USA). For *in vitro* experiments results expressed as mean±standard error of the mean (SEM) and analyzed using ANOVA for multiple comparisons followed where appropriate by paired Student's t tests for paired comparisons. Differences between normal and asthmatic groups were analysed using Mann Whitney tests and correlations using Spearman's rank correlation.
