# Correction: The Role of IL-15 Deficiency in the Pathogenesis of Virus-Induced Asthma Exacerbations

**DOI:** 10.1371/annotation/43a4a197-1739-4561-8b8d-b13cd6d7009f

**Published:** 2012-04-03

**Authors:** Vasile Laza-Stanca, Simon D. Message, Michael R. Edwards, Hayley L. Parker, Mihnea T. Zdrenghea, Tatiana Kebadze, Onn M. Kon, Patrick Mallia, Luminita A. Stanciu, Sebastian L. Johnston

The following information was missing from the funding section: This work was funded in part by the MoRIAE European Research Council FP7 Advanced Investigator award to SLJ, Grant number 233015.

